# The impact of COVID-19 pandemic on sleep duration and mental health among middle school students: a 3-year cross-sectional survey

**DOI:** 10.3389/fpubh.2025.1482309

**Published:** 2025-01-29

**Authors:** Kai-yun Dou, Ru-bao Dong, Chun-long Gan

**Affiliations:** ^1^School of Physical Education, Guizhou Normal University, Guiyang, China; ^2^Guizhou Institute of Sport Science, Guiyang, China

**Keywords:** SARS-CoV-2, epidemic prevention and control, middle school student, sleep duration, mental health

## Abstract

**Background:**

Since the outbreak of COVID-19 in late 2019, and until the Chinese government downgraded the pandemic control measures to Category B management in January 2023, various epidemic prevention measures were implemented across regions based on the local spread of the virus. Correspondingly, educational formats shifted between online and offline teaching according to the pandemic situation. Changes in daily life and teaching methods, along with the high initial mortality rate of COVID-19, have had varying degrees of negative impact on the mental health (MH) of adolescents.

**Purposes:**

This study aimed to explore the impact of the COVID-19 pandemic status on the sleep duration (SD) and MH of adolescents, investigate the relationship between SD and MH in middle school students, and evaluate the protective effect of SD on MH.

**Methods:**

Using a convenient sampling method, 6 junior high schools and 3 senior high schools in Guiyang City, China, were selected. Random samples from two classes per grade in each school were chosen for the survey. The study was conducted annually for three consecutive years using the Mental Health Inventory of Middle-school students (MMHI-60) scale and a self-designed questionnaire. Data were analyzed using IBM SPSS version 26.0 software, employing ANOVA, cross-tabulation, and generalized linear models to examine the relationship between SD and MH scores.

**Results:**

The SD and MH scores of middle school students in 2021, 2022, and 2023 were 436.41 ± 71.21 min/day, 423.69 ± 61.71 min/day, and 445.26 ± 65.04 min/day (F = 41.44, *p* < 0.001), 1.72 ± 0.63, 1.87 ± 0.73, and 1.79 ± 0.67 (F = 18.31, *p* < 0.001). The SD compliance rates were 34.1%, 23.9%, and 33.2%, and the MH problem detection rates were 27.9%, 36.4%, and 33.4%, respectively; there is a significant correlation between SD and MH scores among middle school students (*P* < 0.001); the protective effects of SD compliance on MH were 1.859 times, 2.156 times, and 1.516 times higher than those of SD non-compliance (all *p* < 0.001).

**Conclusion:**

Meeting the SD standard is a protective factor for adolescent MH. The COVID-19 pandemic and its control measures have had a severe and long-term negative impact on both SD and MH in middle school students, with differences observed between genders and academic levels. The greater the severity of the pandemic and the stricter the control measures, the more significant the negative impact on SD and MH etc.

## 1 Introduction

According to the World Health Organization (WHO), by the end of August 2023, the global cumulative number of COVID-19 infections had reached 770,085,713, with a total of 6,956,173 deaths reported (1). Some studies have suggested that during the COVID-19 pandemic, in addition to the direct threat posed by COVID-19 infection, people's health was also influenced by the policies implemented to mitigate the spread of the virus (2). The restrictions on mobility, daily activities, and social interactions imposed by pandemic control measures (3–5) led to significant changes in people's lifestyles. Such drastic lifestyle changes became a major source of stress (6, 7). Moreover, children and adolescents were particularly affected by the restrictive measures during the pandemic (8), with sleep being one of the areas where the health of young people was most severely impacted (9). Persistent sleep problems can have serious consequences (10). Insufficient sleep among middle school students is a global issue (11, 12), and the situation is particularly severe in China (13). Short sleep duration (SD) during adolescence has been linked to poor physical health, mental health (MH) problems, and academic underachievement (14). The impact of the COVID-19 pandemic on sleep may involve various mechanisms, including the release of cytokines, neuroinflammation, changes in daily life (such as social distancing, quarantine, lockdown measures, and economic changes), and immune regulation (15). Sleep plays a crucial role in maintaining balance within the body, supporting the immune system, cognitive function, neural plasticity, memory, energy balance, macromolecule biosynthesis, and metabolic regulation (16, 17). Although most studies suggest that adolescents experienced longer SD during the COVID-19 pandemic (18), the findings regarding the effects of the pandemic on adolescent sleep are inconsistent (19). At the same time, the negative impact of the COVID-19 pandemic on the MH of children and adolescents has been even more pronounced (20, 21).

China's success in containing the COVID-19 virus has largely been attributed to the rapid implementation of isolation and quarantine measures, which were also adopted by other countries (22). With the widespread distribution of COVID-19 vaccines and a decrease in its mortality rate, the Chinese government announced on January 8, 2023, that COVID-19 would be classified as a Category B infectious disease under the new management guidelines (23) and lifted the social control measures related to the virus. During the pandemic, various public health measures were taken to further curb the spread of the virus, including contact tracing, self-isolation, quarantine testing, maintaining social distancing, community activity restrictions, and even lockdowns (24). Before the announcement that COVID-19 would be downgraded to a Category B infectious disease, the Chinese government required regions to implement different control measures based on the local spread of the virus. In areas with active outbreaks, counties, cities, districts, or flags were divided into high-risk and low-risk zones based on the presence of symptomatic cases and asymptomatic carriers. In high-risk zones, lockdown measures were enforced, including “stay-at-home, door-to-door services” and three rounds of nucleic acid testing within the first 3 days after lockdown. Universal testing was conducted, while in low-risk zones, measures such as “personal protection and avoiding gatherings” were applied. Individuals were required to provide proof of a negative nucleic acid test within 48 h if leaving the city. Within these areas, nucleic acid testing was carried out according to local requirements, with reduced outdoor activities, avoidance of gatherings, and personal protective measures during outings. Strict controls on entry into indoor public spaces, including making reservations, staggered access, limiting crowd size, temperature checks, registration, and mandatory mask-wearing, were also enforced (25). In regions without any confirmed COVID-19 cases, routine preventive measures were maintained, and individuals could move freely under conditional circumstances based on their travel history, monitored by a digital tool called the “health code” in WeChat (a Chinese social media app). The health code, also known as the “travel code,” is a software tool used to track an individual's movement and interactions. If an individual had close contact with a confirmed case or had visited a location with a high risk of infection, the code would turn red, indicating the need for physical isolation and medical observation. If the person had been in areas with suspected or potential virus exposure, the code would turn yellow, restricting their social activities and requiring self-isolation and observation for 14 days. If no virus was detected during this period, the health code would turn green, and the individual could resume normal activities. Conversely, if a person tested positive, they would be required to undergo medical isolation, and those in contact with them would need to self-isolate and undergo virus testing (26, 27). This strong control management successfully prevented the widespread pandemic of COVID-19 across China until January 2023, with only sporadic outbreaks in certain regions. Prior to this, most areas in China did not experience scattered cases or concentrated outbreaks of COVID-19. Additionally, they did not experience the three distinct types of pandemic control measures—routine preventive control, emergency response control, and post-control (after the lifting of restrictions)—that characterized the pandemic in many other parts of the world. Corresponding to the control measures, middle school education also shifted between offline and online formats. During offline teaching, students' activities were highly restricted or, in some areas, unrestricted after the easing of measures.

Studies have shown that different populations respond differently to the psychological stress induced by the COVID-19 pandemic and its containment measures (28). The participants in this study were middle school students from Guiyang, Guizhou Province. The first survey was conducted in late October 2021, when there were no reported COVID-19 cases in Guiyang. At that time, people could move freely using the health code system (though in densely populated institutions, such as schools, external visitors were usually restricted to prevent potential virus transmission). The second survey was conducted in late October 2022, shortly after the city had emerged from a large COVID-19 outbreak and the subsequent lockdown measures (29). The third survey was conducted in late October 2023, when, after a year of experiencing COVID-19 outbreaks, the Chinese government had lifted all COVID-19 control measures, and daily life had returned to normal. The participants in all three surveys not only shared the same socio-cultural environment but also experienced the same COVID-19 threat and control policies. However, previous studies examining the impact of COVID-19 on MH have mostly focused on the early phase of the pandemic in the first half of 2020 (30, 31) or have not differentiated between types of COVID-19 control measures (11, 32). These studies were either conducted under the strictest lockdown conditions or in the absence of any control measures, and their results can only be compared to pre-pandemic conditions. Furthermore, differences in demographic characteristics and socio-cultural environments, as well as variations in the COVID-19 threat and control measures experienced by participants, may lead to low homogeneity and comparability of results across different time periods. As a result, there is a need for further research to understand both the short-term and long-term effects of the pandemic on sleep and MH (12), as well as to investigate the sleep and MH impacts of COVID-19 control measures. This study, by comparing data from three different time points during the pandemic, provided a clearer understanding of the impact of varying levels of COVID-19 spread and corresponding control measures on middle school students' SD and MH.

Sleep plays a crucial role in overall health and mental wellbeing and is essential for maintaining and improving physical and psychological health (19, 33). Sleep deprivation can lead to impairments in both physical and mental performance (34). Therefore, this study hypothesizes that SD among middle school students has a protective effect on their MH. The main objective of this study was to explore the influence of the extent of COVID-19 spread and control measures on adolescent MH and SD, investigate the relationship between SD and MH, and assess the protective role of SD in MH. The findings of this study aimed to provide useful insights for promoting adolescent health, particularly in terms of addressing insufficient SD and improving MH.

## 2 Objects and methods

### 2.1 Study participants

In mid-October 2021, a random sampling method was used to select participants from four senior high schools (high-school) and seven junior high schools in Guiyang, Guizhou Province, China. Subsequently, convenience sampling was employed to select entire classes by grade level as the survey participants. To minimize disruption to the students' academic schedule, and with the schools' consent, volunteers were recruited orally, and surveys were administered during rainy days when outdoor physical education classes were canceled. The same schools were selected for the surveys conducted in mid-to-late October 2022 and 2023, but the participants were randomly chosen by entire classes based on weather conditions, meaning that the participant groups for the 3 years were not identical.

### 2.2 Survey instruments

#### 2.2.1 MMHI-60

The Mental Health Inventory of Middle School Students (MMHI-60), developed by Wang et al., consists of 60 items designed specifically to assess the MH of Chinese middle school students. The inventory includes ten factors: obsessive-compulsive symptoms, paranoia, hostility, sensitivity in interpersonal relationships, depression, anxiety, academic stress, maladjustment, emotional instability, and psychological imbalance. It uses a 5-point Likert scale, with higher scores indicating more severe symptoms. A total score and factor-specific mean scores ≥2 indicate mild symptoms. The total score reflects the overall MH status, referred to as psychological state. The reliability of the scale, as measured by test-retest reliability, ranges from 0.716 to 0.905, and the correlations between the total score and the subscales range from 0.7652 to 0.8726. The correlations between the subscales range from 0.4027 to 0.7587 (35). In this study, the internal consistency reliability (Cronbach's α) of the MMHI-60 was 0.970, and the Kaiser-Meyer-Olkin (KMO) measure of sampling adequacy was 0.980 (*p* < 0.05).

#### 2.2.2 A self-made questionnaire

The custom questionnaire collected basic demographic information, including the participant's gender, grade, and age. It also gathered details about sleep patterns, such as the time participants go to bed, the time they wake up in the morning, and the start and end times of their afternoon naps. Since sleep opportunity, as a proxy for SD, is a commonly used method in pediatric sleep research (36), the questionnaire focused on these key sleep-related variables to calculate total SD.

### 2.3 Sampling basis

The sample size calculation formula used in this study was: N = Z^2^ × [P × (1–P)]/E^2^ (37), where N is the sample size, Z is the statistical value for a 95% confidence interval (Z = 1.96), E is the margin of error (E = 0.05), and P is the estimated population proportion (P = 0.5). Thus, the required sample size was approximately 385. Given that middle school and high school students differ significantly in cognitive abilities and academic stress, this study treated middle school and high school students as two distinct groups and conducted separate sampling for each group. Each year, the sample size for both middle school and high school students exceeded 500 participants, ensuring strong representativeness of the sample.

### 2.4 Questionnaire survey

This study was approved by the Academic Ethics Committee of the School of Physical Education, Guizhou Normal University. The survey participants were recruited orally by physical education teachers from the students' respective schools, who also distributed the informed consent forms. The signed consent forms from parents were collected 1 week later, after which the students were eligible to participate in the survey. After randomly selecting the schools where the survey subjects were located in 2021, in order to maintain the same or similar social and cultural attributes of the survey subjects in different years as much as possible, we directly selected these schools for the questionnaire surveys in 2022 and 2023; Similarly, during the survey, students who agree to participate in the questionnaire survey were selected based on weather conditions from classes with physical education classes but cannot attend physical education classes outdoors. On rainy days when outdoor physical education classes were canceled, trained surveyors gathered the participants in classrooms to distribute, complete, and collect the questionnaires. The surveys were administered in late October for 2021 and 2023 and in mid-October 2022.

To ensure an adequate number of participants, we conducted a general recruitment before the survey and obtained parental informed consent. After randomly selecting schools, we communicated with the school principals to ensure that the survey would not disrupt the students' normal learning. The surveyors underwent COVID-19 nucleic acid testing in advance according to local epidemic prevention policies and provided proof of non-infection. During and after the epidemic prevention period, we randomly selected classes that could not hold physical education classes due to rain, and within these classes, students were randomly chosen for the questionnaire survey. The 2022 survey was conducted in mid-October to assess the MH and sleep patterns of the participants during the COVID-19 outbreak and after the resumption of in-person classes. The distribution of survey participants is shown in Table 1. For convenience, the following terminology will be used throughout this paper: 2021 refers to the period of routine control before the COVID-19 outbreak, 2022 refers to the emergency management period during the COVID-19 outbreak, and 2023 refers to the post-pandemic period after the lifting of control measures.

**Table 1:** Overview of annual distribution of survey subjects by grade, age, and gender (*N* = 4,591).

**Year**	**Junior high school students**				**Senior high school students**				**Age**		**Gender**		**Total**
	**7**	**8**	**9**	**Subtotal**	**10**	**11**	**12**	**Subtotal**	**Mean**	**Std**.	**Male**	**Female**	
2021	329	301	267	897	228	218	143	589	14.93	1.76	750	736	1,486
2022	177	258	208	643	300	292	234	826	14.91	1.7	735	734	1,469
2023	461	396	237	1,094	152	235	155	542	14.11	1.75	803	833	1,636

As shown in Table 1, the gender ratio and age of the participants were very similar across the different years. The number of participants was smallest in 2022, while the largest number of participants was in 2023. However, the number of participants in 2021 and 2022 was almost identical. In 2021 and 2023, the number of middle school students was greater than that of high school students, while in 2022, the number of high school students was higher than that of middle school students. The lower number of high school students in 2021 and 2023 can be attributed to their heavy academic workloads and reluctance to participate in surveys they consider unrelated to their studies. In contrast, the higher number of high school students participating in the 2022 survey was likely due to their heightened awareness of MH issues as a result of the COVID-19 outbreak and related control measures.

### 2.5 Data statistics method

IBM SPSS version 26.0 software was used for statistical analysis, primarily employing one-way ANOVA to compare mean values. Cross-tabulation was used to examine differences in SD and MH problem detection rates across the different periods. Comparisons were made between the overall, middle school, and high school student groups' MH status, SD, and major changes during the period of routine control before the COVID-19 outbreak (2021), the emergency management period during the pandemic (2022), and the post-pandemic period after the lifting of control measures (2023). Additionally, a log-binomial generalized linear model (GLM) was used to compare differences in MH problem detection rates between students with adequate and inadequate SD. The significance level was set at *p* < 0.05.

## 3 Results

### 3.1 Characteristics and comparison of SD and MH among middle school students

The average SD for middle school students, including male and female students, gradually decreased as the severity of COVID-19 control measures increased. Even after the lifting of these measures, the average SD did not reach the recommended minimum of 8 h per day. Furthermore, there were significant differences in SD between middle school students, male students, and female students. The MH scores of middle school students were highest during the COVID-19 outbreak period and lowest during the routine control period, with significant differences observed between the MH scores across different periods.

For junior high school students, the average SD was longest during the routine control period and shortest during the COVID-19 outbreak period, with significant differences in SD between these periods. None of the students met the recommended 8 h of sleep per day. MH scores for junior high school students were highest after the lifting of pandemic control measures and lowest during the routine control period, with significant differences in MH scores observed across periods.

For senior high school students, the average SD was lowest during the routine control period and highest after the lifting of pandemic control measures, with significant differences between these periods. However, the average SD was still below 7 h per day. MH scores for senior high school students were lowest during the routine control period and highest during the COVID-19 outbreak period, with significant differences in MH scores between these periods (Table 2).

**Table 2:** Overview of MH and SD scores for middle school students.

**Group**	**Variable**	**Year**	**N**	**Mean**	**Std. D**.	**Std. E**.	**95% CI for Mean**		**ANOVA**	
							**LB**	**UB**	**F**	**p**
Middle school students	SD	2021	1,486	436.41	71.21	1.85	432.79	440.04	41.44	0.000
		2022	1,469	423.69	61.71	1.61	420.53	426.85		
		2023	1,636	445.26	65.04	1.61	442.10	448.41		
	MH	2021	1,486	1.72	0.63	0.02	1.69	1.76	18.31	0.000
		2022	1,469	1.87	0.73	0.02	1.84	1.91		
		2023	1,636	1.79	0.67	0.02	1.76	1.82		
Male	SD	2021	750	439.27	72.81	2.66	434.05	444.49	19.79	0.000
		2022	735	426.14	63.18	2.33	421.57	430.72		
		2023	803	447.77	66.64	2.35	443.16	452.39		
	MH	2021	750	1.69	0.62	0.02	1.65	1.74	8.18	0.000
		2022	735	1.84	0.75	0.03	1.78	1.89		
		2023	803	1.75	0.67	0.02	1.70	1.80		
Female	SD	2021	736	433.51	69.47	2.56	428.48	438.53	21.98	0.000
		2022	734	421.23	60.14	2.22	416.87	425.59		
		2023	833	442.83	63.42	2.20	438.52	447.14		
	MH	2021	736	1.75	0.64	0.02	1.71	1.80	10.20	0.000
		2022	734	1.91	0.71	0.03	1.86	1.97		
		2023	833	1.83	0.67	0.02	1.78	1.88		
Junior high school students	SD	2021	897	465.56	63.11	2.11	461.42	469.69	3.20	0.041
		2022	643	457.52	58.79	2.32	452.97	462.07		
		2023	1,094	463.21	63.59	1.92	459.44	466.98		
	MH	2021	897	1.65	0.58	0.02	1.61	1.68	6.07	0.002
		2022	643	1.67	0.65	0.03	1.62	1.72		
		2023	1,094	1.74	0.66	0.02	1.70	1.78		
Senior high school students	SD	2021	589	392.03	58.90	2.43	387.27	396.80	15.05	0.000
		2022	826	397.35	49.99	1.74	393.94	400.76		
		2023	542	409.02	51.47	2.21	404.68	413.36		
	MH	2021	589	1.84	0.68	0.03	1.79	1.90	13.99	0.000
		2022	826	2.03	0.75	0.03	1.98	2.09		
		2023	542	1.89	0.69	0.03	1.83	1.95		

LB, Lower Bound; UB, Upper Bound, the same below.

*Post-hoc* multiple comparisons indicated that the significant differences in SD among middle school students, male and female students, were most pronounced during the COVID-19 outbreak period, when SD was significantly lower than during other periods. After the lifting of pandemic control measures, SD was significantly higher than during the routine control period. As for MH scores, significant differences were observed with higher MH scores during the COVID-19 period compared to other periods. MH scores after the lifting of control measures were significantly higher than during the routine control period. During the COVID-19 period, male students' MH scores were significantly higher than during the routine control period. Female students had significantly higher MH scores during the COVID-19 outbreak period compared to other periods.

For junior high school students, SD during the routine control period was significantly higher than during the COVID-19 outbreak period. After the lifting of pandemic control measures, MH scores were significantly higher than during the routine control period. For senior high school students, SD after the lifting of control measures was significantly higher than during other periods. MH scores during the COVID-19 period were significantly higher than during other periods (Table 3).

**Table 3:** Summary of multiple comparisons (Dunnett T3).

**Group**	**Variable**	**(I) year**	**(J) year**	**(I-J) mean difference**	**Std. E**	***p***	**95% CI**	
							**LB**	**UB**
Students	SD	2021	2022	12.73^*^	2.45	0.000	6.87	18.58
		2023	2021	8.84^*^	2.45	0.001	2.99	14.70
			2022	21.57^*^	2.28	0.000	16.13	27.01
	MH	2022	2021	0.15^*^	0.03	0.000	0.09	0.21
			2023	0.08^*^	0.03	0.003	0.02	0.14
		2023	2021	0.07^*^	0.02	0.011	0.01	0.12
Male	SD	2021	2022	13.13^*^	3.54	0.001	4.67	21.58
		2023	2021	8.51^*^	3.55	0.049	0.02	16.99
			2022	21.63^*^	3.31	0.000	13.72	29.55
	MH	2022	2021	0.14^*^	0.04	0.000	0.06	0.23
Female	SD	2021	2022	12.28^*^	3.39	0.001	4.32	20.23
		2023	2021	9.33^*^	3.37	0.016	1.41	17.24
			2022	21.60^*^	3.12	0.000	14.27	28.93
	MH	2022	2021	0.16^*^	0.04	0.000	0.08	0.24
			2023	0.08^*^	0.04	0.045	0.00	0.17
Junior	SD	2021	2022	8.03^*^	3.13	0.031	0.55	15.52
	MH	2023	2021	0.10^*^	0.03	0.002	0.03	0.16
Senior	SD	2023	2021	16.99^*^	3.28	0.000	9.14	24.84
			2022	11.67^*^	2.81	0.000	4.94	18.40
	MH	2022	2021	0.19^*^	0.04	0.000	0.10	0.28
			2023	0.14^*^	0.04	0.001	0.05	0.24

^*^The mean difference is significant at the 0.05 level; (I-J) = Mean Difference (I-J); students = Middle School students, Junior = Junior High School Students, Senior = Senior High School Students, the same below.

### 3.2 Changes in the detection rate of SD insufficiency and MH issues among middle school students

To more accurately understand the relationship between the SD compliance rate and the detection rate of MH issues in middle school students, SD was categorized into two groups according to the National Sleep Foundation's recommendation for teenagers (8–10 h per day) (38). Students with SD < 8 h per day were classified as “SD below standard” (SD < 8), and those with SD ≥ 8 h per day were classified as “SD standard” (SD ≥ 8). Simultaneously, based on the MMHI-60 scoring criteria, MH scores ≥ 2 were categorized as “abnormal” (indicating MH issues), and MH scores < 2 as “normal” (indicating good MH).

During the COVID-19 outbreak period, middle school students, as well as male and female students, exhibited the lowest SD compliance rate, while the highest compliance rate was observed during the routine control period. The difference in SD compliance rates between the routine control and post-pandemic periods was < 1%. The overall SD compliance rate for middle school students was below 35%, with male students showing slightly higher rates than female students, although the rates remained under 40%.

Junior high school students had the highest SD compliance rate during the COVID-19 outbreak period and the lowest rate after the lifting of pandemic control measures. The SD compliance rate for junior high school students exceeded 40%, with the rate approaching 50% during periods of active pandemic control measures. Senior high school students, on the other hand, had the lowest SD compliance rate during the COVID-19 outbreak period and the highest after the lifting of pandemic control measures. In the COVID-19 outbreak period, the SD compliance rate for senior high school students was under 5%, with the rate slightly above 10% in other periods.

For MH issues, middle school students, male and female students, exhibited the highest detection rates during the COVID-19 outbreak period, with the lowest rates observed during the routine control period. Female students had slightly higher MH issue detection rates than male students in the same period. Except for the routine control period, the detection rate of MH issues in all other periods exceeded 30%.

For junior high school students, the detection rate of MH issues was highest after the lifting of pandemic control measures, reaching 30%. In contrast, the lowest rate was observed during the COVID-19 outbreak period. Among senior high school students, the detection rate of MH issues reached its peak during the COVID-19 period, at 46.7%, and remained above 40% after the lifting of pandemic control measures. The lowest rate of MH issues was observed during the routine control period.

Significant differences in SD compliance rates and MH issue detection rates were observed between years for all student groups (Table 4). However, the specific significance of differences in SD and MH scores across periods remains unclear.

**Table 4:** Summary of SD compliance rate and MH problem detection rate among middle school students.

**Group**	**Year**	**Compliance rate of SD**	**Pearson Chi-Square**		**Problem detection rate of MH**	**Pearson Chi-Square**	
			**Value**	**p(2-sided)**		**Value**	**p(2-sided)**
All samples	2021	34.1	44.677	< 0.001	27.9	25.591	< 0.001
	2022	23.9			36.4		
	2023	33.2			33.4		
Male	2021	36.8	31.489	< 0.001	26.5	12.474	0.002
	2022	24.6			35		
	2023	36			31.4		
Female	2021	31.3	14.642	0.001	29.2	13.229	0.001
	2022	23.2			37.9		
	2023	30.5			35.4		
Junior high school student	2021	49.5	9.072	0.011	23.4	14.992	0.001
	2022	49.6			23.2		
	2023	43.6			30		
Senior high school student	2021	10.5	36.254	0.000	34.6	20.919	0.000
	2022	3.9			46.7		
	2023	12.2			40.4		

The significant differences in SD across different years indicated that SD during the COVID-19 period was significantly lower than during the routine control and post-pandemic periods. MH scores during the routine control period were significantly higher than those during the COVID-19 outbreak and post-pandemic periods, while no notable differences in SD and MH scores were observed in other periods.

For male and female students, significant differences in SD were only observed during the COVID-19 outbreak period, where SD was significantly lower than during the routine control and post-pandemic periods. Additionally, MH scores during the routine control period were significantly higher than during the COVID-19 and post-pandemic periods.

For both junior and senior high school students, SD after the lifting of pandemic control measures was significantly higher than during the routine control and COVID-19 outbreak periods. MH scores after the lifting of pandemic control measures were also significantly higher than during the COVID-19 outbreak and routine control periods. Moreover, senior high school students had significantly higher SD and MH scores during the routine control period compared to the COVID-19 outbreak period (Table 5).

**Table 5:** Specific years with significant differences in SD compliance rate and MH problem detection rate (summary).

**Group**	**Dependent variable**	**Year**	**Year**	**Value**	**df**	**Asymptotic Significance (2-sided)**
All samples	SD	2022	2021	37.014	1	< 0.001
			2023	32.630	1	< 0.001
	MH	2021	2022	24.824	1	< 0.001
			2023	12.603	1	< 0.001
Male	SD	2022	2021	25.825	1	< 0.001
			2023	23.354	1	< 0.001
	MH	2021	2022	12.406	1	< 0.001
			2023	4.492	1	0.034
Female	SD	2022	2021	12.141	1	< 0.001
			2023	10.626	1	0.001
	MH	2021	2022	12.372	1	< 0.001
			2023	7.741	1	0.005
Junior high school student	SD	2023	2021	11.182	2	0.004
			2022	25.377	2	< 0.001
	MH	2023	2022	26.392	2	< 0.001
			2021	21.758	2	< 0.001
Senior high school student	SD	2023	2022	34.029	2	< 0.001
			2021	22.475	2	< 0.001
		2021	2022	24.533	1	< 0.001
	MH	2023	2022	11.936	2	0.003
			2021	14.086	2	0.001
		2021	2022	20.694	1	< 0.001

### 3.3 Impact of SD on MH in middle school students

Studies have shown that when the prevalence is relatively high, the prevalence ratio (PR) is a better measure of the association between exposure and disease than the odds ratio (OR) (39). Additionally, the GLM is an extension of the standard linear model, where the response variable no longer follows just a normal distribution but rather one from the exponential family of distributions, greatly expanding the standard linear model (40). In cross-sectional studies, when the outcome incidence is high (e.g., >10%), using a logistic regression model to calculate the OR may overestimate the strength of the association between the outcome and factors. In such cases, the log-binomial regression model should be appropriately used to calculate the PR and explore the factors influencing the occurrence of outcomes more scientifically (41). To more accurately understand the relationship between SD compliance rates and the detection of MH issues, we divided students into two groups: SD compliant vs. SD non-compliant, and MH problem vs. MH healthy, and conducted log-binomial GLM analysis. Specifically, we categorized students' SD into SD non-compliant and SD compliant groups as independent variables, and their MH scores into MH problem and MH healthy groups as dependent variables. The log-binomial GLM was used to compare the PR across different periods, further exploring whether students with non-compliant SD were more likely to experience MH issues compared to those with compliant SD.

The correlation coefficients between SD and MH scores for middle school students were 0.239 (2021), 0.249 (2022), and 0.218 (2023), all of which were statistically significant (*P* < 0.001). The highest correlation coefficient was observed during the COVID-19 outbreak period, and the lowest during the post-pandemic period after control measures were lifted.

The β-values for middle school students, male, and female students were highest during the COVID-19 outbreak period and lowest after the lifting of pandemic control measures, with all *P*-values being < 0.01. For junior high school students, the β-value was highest during the routine control period and lowest after the lifting of pandemic measures, with all *P*-values being < 0.001. For senior high school students, the β-value was highest during the routine control period and lowest during the COVID-19 outbreak period, with *P*-values for all periods being < 0.05, although no significant findings were observed. These findings suggest that SD compliance is a protective factor for MH in middle school students, including male and female students and junior high school students. However, SD compliance is not an effective protective factor for the MH of senior high school students (Table 6).

**Table 6:** Parameter estimates.

**Group**		**Parameter**	**B**	**Std.E**	**95% Wald CI**		**Hypothesis Test**			**Exp(B)**	**95% Wald CI for Exp(B)**	
					**Lower**	**Upper**	**Wald**χ^2^	**df**	**Sig**.		**Lower**	**Upper**
Stuednts	2021	(Intercept)	−1.727	0.096	−1.91	−1.54	326.398	1	0.000	0.178	0.15	0.21
		[SD not = 2.00]	0.620	0.106	0.41	0.83	34.310	1	0.000	1.859	1.51	2.29
		[SD std. = 1.00]	0a							1		
	2022	(Intercept)	−1.641	0.109	−1.85	−1.43	227.193	1	0.000	0.194	0.16	0.24
		[SD not = 2.00]	0.768	0.114	0.54	0.99	45.048	1	0.000	2.156	1.72	2.70
		[SD std. = 1.00]	0a							1		
	2023	(Intercept)	−1.392	0.075	−1.54	−1.25	348.056	1	0.000	0.249	0.21	0.29
		[SD not = 2.00]	0.416	0.084	0.25	0.58	24.470	1	0.000	1.516	1.29	1.79
		[SD std. = 1.00]	0a							1		
Male	2021	(Intercept)	−1.859	0.140	−2.13	−1.58	176.065	1	0.000	0.156	0.12	0.21
		[SD not = 2.00]	0.748	0.155	0.44	1.05	23.368	1	0.000	2.112	1.56	2.86
		[SD std. = 1.00]	0a							1		
	2022	(Intercept)	−1.940	0.181	−2.30	−1.58	114.315	1	0.000	0.144	0.10	0.21
		[SD not = 2.00]	1.066	0.188	0.70	1.43	32.025	1	0.000	2.903	2.01	4.20
		[SD std. = 1.00]	0a							1		
	2023	(Intercept)	−1.418	0.104	−1.62	−1.21	185.722	1	0.000	0.242	0.20	0.30
		[SD not = 2.00]	0.380	0.120	0.14	0.61	10.031	1	0.002	1.462	1.16	1.85
		[SD std. = 1.00]	0a							1		
Female	2021	(Intercept)	−1.588	0.130	−1.84	−1.33	148.949	1	0.000	0.204	0.16	0.26
		[SD not = 2.00]	0.485	0.145	0.20	0.77	11.269	1	0.001	1.625	1.22	2.16
		[SD std. = 1.00]	0a							1		
	2022	(Intercept)	−1.398	0.134	−1.66	−1.14	109.039	1	0.000	0.247	0.19	0.32
		[SD not = 2.00]	0.527	0.143	0.25	0.81	13.615	1	0.000	1.694	1.28	2.24
		[SD std. = 1.00]	0a							1		
	2023	(Intercept)	−1.363	0.107	−1.57	−1.15	162.272	1	0.000	0.256	0.21	0.32
		[SD not = 2.00]	0.440	0.119	0.21	0.67	13.744	1	0.000	1.552	1.23	1.96
		[SD std. = 1.00]	0a							1		
Junior	2021	(Intercept)	−1.805	0.107	−2.02	−1.60	284.749	1	0.000	0.164	0.13	0.20
		[SD not = 2.00]	0.609	0.129	0.36	0.86	22.459	1	0.000	1.839	1.43	2.37
		[SD std. = 1.00]	0a							1		
	2022	(Intercept)	−1.814	0.127	−2.06	−1.57	204.424	1	0.000	0.163	0.13	0.21
		[SD not = 2.00]	0.608	0.153	0.31	0.91	15.848	1	0.000	1.837	1.36	2.48
		[SD std. = 1.00]	0a							1		
	2023	(Intercept)	−1.485	0.085	−1.65	−1.32	308.031	1	0.000	0.226	0.19	0.27
		[SD not = 2.00]	0.454	0.100	0.26	0.65	20.446	1	0.000	1.575	1.29	1.92
		[SD std. = 1.00]	0a							1		
Senior	2021	(Intercept)	−1.294	0.207	−1.70	−0.89	39.214	1	0.000	0.274	0.18	0.41
		[SD not = 2.00]	0.258	0.215	−0.16	0.68	1.441	1	0.230	1.294	0.85	1.97
		[SD std. = 1.00]	0a							1		
	2022	(Intercept)	−0.693	0.177	−1.04	−0.35	15.374	1	0.000	0.500	0.35	0.71
		[SD not = 2.00]	−0.070	0.181	−0.42	0.28	0.152	1	0.697	0.932	0.65	1.33
		[SD std. = 1.00]	0a							1		
	2023	(Intercept)	−0.894	0.148	−1.18	−0.60	36.504	1	0.000	0.409	0.31	0.55
		[SD not = 2.00]	−0.014	0.158	−0.32	0.30	0.008	1	0.929	0.986	0.72	1.34
		[SD std. = 1.00]	0a							1		

SD not, SD not up to standard; SD std., SD standard.

## 4 Discussion

The SD of middle school students decreased significantly as COVID-19 control measures became stricter, with an average SD of < 6.5 h per day. This trend in SD changes is similar to that observed in South Korean adolescents, although the SD of Chinese middle school students remain higher than that of their South Korean counterparts (42). The primary reason for this decline is likely the lockdown measures implemented during the COVID-19 outbreak, which may have altered adolescents' sleep patterns (9). Remote online learning during the pandemic led to changes in adolescents' daily routines (43), and the difficulty in initiating and maintaining sleep, as well as delayed sleep/wake behaviors, increased (44). However, this finding contrasts with studies suggesting that online learning, by reducing commuting time, allowed adolescents to get more sleep (45). Instead, it is more likely that during the COVID-19 pandemic, children and adolescents spent more time online or on social media platforms, which could have negatively affected their sleep (46).

Research has shown that adolescents who do not get enough sleep tend to have poorer MH outcomes (42). In this study, middle school students had the lowest MH scores during the normalized control period, the highest scores during the COVID-19 outbreak, and did not fully return to baseline MH levels nearly 1 year after the lifting of COVID-19 restrictions. The high MH scores during the outbreak, despite reduced SD, are consistent with findings suggesting that individuals with less sleep than the recommended duration face a higher risk of MH issues (47). During the same period, boys had slightly more sleep than girls on average, but girls had higher MH scores and a slightly higher rate of MH issues. The proportion of middle school students achieving the recommended 8 h of sleep per day was less than one-third, which aligns with the findings in the “China Sleep Research Report 2023” (13). Due to academic pressure, high levels of stress, and other factors, insufficient and poor-quality sleep has become a norm for adolescents (29). The trends in MH problem detection rates suggest that the COVID-19 outbreak had a significant impact on the MH of middle school students, and there was no significant recovery in the post-pandemic period. Comparisons with other studies show that the MH problem detection rate before the pandemic is consistent with the findings of Dong and Dou (48). However, the detection rate during the COVID-19 outbreak was lower than the 44.9% prevalence of MH issues among adolescents during the pandemic (49). MH problem rates during the COVID-19 outbreak, before, and after the pandemic were consistently higher than the 26.3% rate found before the pandemic (49), and much higher than the global prevalence of approximately 14% among adolescents (aged 10–19) with MH issues (50). Yet, the MH problem detection rate during the COVID-19 outbreak in this study was lower than the findings of Liang et al. (51), and the incidence of depression among middle school students was lower than the global rates of anxiety (50.9%) and depression (48.3%) in the general population (52). These findings suggest that while lockdown measures may have had a negative impact on MH (53), the Chinese government's provision of psychological support to schools and vulnerable groups during the pandemic (54) likely helped mitigate some of the MH issues among middle school students. Moreover, the COVID-19 outbreak in Guiyang, where the study took place, only occurred in September 2022, and by this time, people were no longer as fearful of COVID-19 as they were at the onset of the pandemic. However, it is important to note that the MH detection rates may also be influenced by variations in measurement tools, detection criteria, and timing of assessments (55). The MH scores of middle school students remained higher nearly 1 year after the lifting of pandemic control measures compared to the normalized control period, indicating that the negative impact of the COVID-19 outbreak on certain mental health issues may be long-lasting. This also suggests that, in the post-pandemic era, other factors may continue to negatively affect the mental health of middle school students. The higher MH scores of girls and high school students compared to boys and junior high school students could be attributed to differences in gender, age, and educational demands (56). Since sleep deprivation continues to be associated with MH problems (57), it is not surprising that the detection rate of MH issues is higher among girls and high school students compared to boys and junior high students. Those who sleep 6 h or less are more likely to experience MH issues than those who sleep 8 h or more (42). Therefore, it is not surprising that the detection rate of MH issues is higher among female students and high school students compared to male students and junior high school students.

The results of the study showed that the development trends of SD and MH scores for junior high school students differed from those of senior high school students. For junior high students, the SD was the longest during the normalized control period and the shortest during the COVID-19 outbreak. The average MH score during the normalized control period was the lowest, while it was the highest after the lifting of the pandemic restrictions. The changes in SD and MH scores among junior high students can likely be attributed to several factors: Firstly, in July 2021, the General Office of the Communist Party of China and the State Council issued the “Opinions on Further Reducing the Homework Burden and Extracurricular Training Burden of Students in the Compulsory Education Stage” (58). This policy led to a reduction in students' homework burden, lowered subjective learning pressure, and decreased the proportion of students attending extracurricular tutoring programs. Secondly, during the normalized control period, in order to prevent the spread of the pandemic, the Ministry of Education mandated the closure of extracurricular training institutions (59). This alleviated the burden on junior high students from participating in various types of extracurricular activities, effectively reducing their academic load. Especially during the COVID-19 outbreak, online learning, which eliminated commuting time, and the inability of teachers to directly supervise or monitor students' completion of homework, also helped reduce academic pressure for junior high students. However, concerns over COVID-19—such as fear of infection for themselves or their families, anxiety over quarantine, and spending excessive time online following the developments of the pandemic (with 92% of Chinese minors owning smartphones and one-third of preschool children being internet users) (60)—consumed a lot of junior high students' time and increased their psychological burden. This contrasts with findings such as those of a study indicating that the prevalence of MH issues among junior high students was significantly higher during the COVID-19 outbreak compared to pre-pandemic times (61). However, the MH scores for junior high students did not show a significant increase after the outbreak of COVID-19. This could be due to several reasons: after more than 2 years of the pandemic, most individuals had been vaccinated against COVID-19 (62), and psychological support provided by the government and educational departments helped alleviate some students' MH issues (63). Additionally, the fatality rate of COVID-19 had decreased to a lower level, and people were no longer as fearful of the virus as before. After the lifting of pandemic control measures, MH scores for junior high students continued to rise. This may be due to the reopening of various educational and training institutions after the pandemic restrictions were lifted, which meant that students had to resume attending various training programs. Therefore, it can be reasoned that the stress from academic burdens on junior high students has a more significant negative impact on physical activity and MH than the pandemic itself and its corresponding social control measures.

Additionally, we also found that for senior high school students, SD was highest after the lifting of the pandemic control measures and lowest during the normalized control period. MH scores were highest during the COVID-19 outbreak and lowest during the normalized control period. After the lifting of pandemic restrictions, SD in senior high school students significantly increased, but the proportion of students achieving 8 h of sleep per day was just over 12%. During the COVID-19 outbreak, although the average SD increased slightly, the SD compliance rate was the lowest, below 4%. The outbreak of COVID-19 had a significant negative impact on the MH of senior high school students, with the detection rate of MH problems exceeding 46%. Even after the lifting of pandemic restrictions, the detection rate remained above 40%. This indicates that the outbreak of COVID-19 and the corresponding control measures had a widespread negative impact on both the MH and SD of senior high school students (Note: Senior high school education in China is not part of compulsory education). Combining the increase in MH problem detection rates in junior high school students after the lifting of pandemic controls suggests that, in the post-pandemic era, families, schools, and society may not have paid sufficient attention to psychological guidance for students or implemented effective measures to help students recover their MH.

Log-binomial GLM analysis indicated that, compared with students who did not meet SD standards, those who met the SD standards showed better MH. The β values for middle school students, male and female students, were highest during the COVID-19 period and lowest after the lifting of the pandemic restrictions. For junior high school students, the β values were highest during the normalized control period and lowest after the lifting of the pandemic controls. Senior high school students, however, showed no protective effect of SD on MH. This suggests that meeting SD standards serves as a protective factor for the MH of middle school students, with the protective effect being more prominent when external factors directly affect students. However, for senior high school students who experienced significant sleep deprivation, SD did not provide any protective benefit for their MH.

Although the participants in this study are not the same fixed group, the accuracy of data comparison between different years may be reduced. In fact, under the strict pandemic control measures implemented by the Chinese government, it is not guaranteed that COVID-19 would necessarily have spread to a specific area or targeted a specific group of participants. It is also difficult to predict that a certain age group can experience the direct and indirect threats of the COVID-19 pandemic, varying degrees of strict social epidemic prevention and control measures, and the lifting of social prevention and control measures. Therefore, conducting a continuous cross-sectional survey of middle school students with the same social environment and cultural atmosphere to compare the impact of the COVID-19 pandemic and corresponding epidemic prevention and control measures on the physical activity level and mental health of middle school students, as well as the recovery status of physical activity level and mental health of middle school students after the lifting of epidemic prevention and control, also has important reference value for promoting the physical and mental health of middle school students in the event of major disasters.

### 4.1 Limitations

This study is based on cross-sectional data, which may be subject to recall bias. It did not account for whether the participants had family members infected with COVID-19. Although the MMHI-60 is one of the most commonly used MH scales in China, it is not a professional screening tool for MH issues, so the detection rate of MH problems may be subject to some degree of error. The survey sample was drawn from a single city, and factors such as the local economic, educational, and cultural environment may limit the generalizability of the findings. Moreover, since the participants were not all from the same group, longitudinal comparisons may have certain biases. Additionally, differences in the number of junior and senior high school students in the sample may have influenced the research results.

## 5 Conclusion and recommendations

The COVID-19 pandemic and its control measures have had a significant and long-lasting negative impact on the SD and MH of middle and high school students. The stricter the pandemic control measures, the more severe the negative effects on students' SD and MH. These negative impacts vary by gender and educational stage. The academic burden on junior high students was relatively lighter, and the pandemic's negative impact was mainly on their SD, while the academic burden on senior high school students was heavier, and the negative impact of the pandemic was more pronounced on their MH. Female students were more negatively affected by both SD and MH issues compared to male students. Meeting the recommended SD standards serves as a protective factor for students' MH, and this protective effect is more pronounced when external factors interfere. Families, schools, and society should pay comprehensive attention to students' MH after the lifting of pandemic restrictions, providing timely psychological support for students at risk of MH issues. At the same time, efforts should be made to create conditions that enable students to achieve the recommended SD.

## Data Availability

The original contributions presented in the study are included in the article/supplementary material, further inquiries can be directed to the corresponding author.
